# Diffusion Weighted Imaging and T2 Mapping Detect Inflammatory Response in the Renal Tissue during Ischemia Induced Acute Kidney Injury in Different Mouse Strains and Predict Renal Outcome

**DOI:** 10.3390/biomedicines9081071

**Published:** 2021-08-23

**Authors:** Robert Greite, Katja Derlin, Dagmar Hartung, Rongjun Chen, Martin Meier, Marcel Gutberlet, Bennet Hensen, Frank Wacker, Faikah Gueler, Susanne Hellms

**Affiliations:** 1 Nephrology, Hannover Medical School, 30625 Hannover, Germany; Greite.Robert@mh-hannover.de (R.G.); Chen.Rongjun@mh-hannover.de (R.C.); gueler.faikah@mh-hannover.de (F.G.); 2 Radiology, Hannover Medical School, 30625 Hannover, Germany; Derlin.Katja@mh-hannover.de (K.D.); Hartung.Dagmar@mh-hannover.de (D.H.); Gutberlet.Marcel@mh-hannover.de (M.G.); Hensen.Bennet@mh-hannover.de (B.H.); Wacker.Frank@mh-hannover.de (F.W.); 3 Laboratory Animal Science, Hannover Medical School, 30625 Hannover, Germany; Meier.Martin@mh-hannover.de

**Keywords:** diffusion weighted imaging, T2 mapping, functional MRI, acute kidney injury, ischemia reperfusion injury

## Abstract

To characterize ischemia reperfusion injury (IRI)-induced acute kidney injury (AKI) in C57BL/6 (B6) and CD1-mice by longitudinal functional MRI-measurement of edema formation (T2-mapping) and inflammation (diffusion weighted imaging (DWI)). IRI was induced with unilateral right renal pedicle clamping for 35min. 7T-MRI was performed 1 and 14 days after surgery. DWI (7 b-values) and multiecho TSE sequences (7 TE) were acquired. Parameters were quantified in relation to the contralateral kidney on day 1 (d1). Renal MCP-1 and IL-6-levels were measured by qPCR and serum-CXCL13 by ELISA. Immunohistochemistry for fibronectin and collagen-4 was performed. T2-increase on d1 was higher in the renal cortex (127 ± 5% vs. 94 ± 6%, *p* < 0.01) and the outer stripe of the outer medulla (141 ± 9% vs. 111 ± 9%, *p* < 0.05) in CD1, indicating tissue edema. Medullary diffusivity was more restricted in CD1 than B6 (d1: 73 ± 3% vs. 90 ± 2%, *p* < 0.01 and d14: 77 ± 5% vs. 98 ± 3%, *p* < 0.01). Renal MCP-1 and IL-6-expression as well as systemic CXCL13-release were pronounced in CD1 on d1 after IRI. Renal fibrosis was detected in CD1 on d14. T2-increase and ADC-reduction on d1 correlated with kidney volume loss on d14 (r = 0.7, *p* < 0.05; r = 0.6, *p* < 0.05) and could serve as predictive markers. T2-mapping and DWI evidenced higher susceptibility to ischemic AKI in CD1 compared to B6.

## 1. Introduction

Acute kidney injury (AKI) due to ischemia reperfusion injury (IRI) is common in many clinical situations including cardiac surgery and hemodynamic instability [1]. AKI is associated with an increased risk for progression to chronic kidney disease (CKD) and poor patient outcome [2,3]. Several other risk factors for the development and aggravation of AKI have been identified such as hypertension, diabetes or drug related side-effects [4,5].

C57BL/6 (B6) mice are a commonly used mouse strain for IRI-induced AKI studies and background for many genetic knockout models in renal IRI [6,7]. However, strain-specific differences in the susceptibility to renal IRI have been described [8,9]. A recent study used functional MRI in CD1 mice and found that they exhibit pronounced renal perfusion impairment following IRI compared to B6 [9]. In addition, CD1 mice had blood pressure elevation and glomerular injury following partial nephrectomies [10].

Functional magnetic resonance imaging (MRI) provides promising techniques for the evaluation of renal pathophysiology [11,12,13,14] and offers the possibility to assess multiple parameters noninvasively without the use of contrast agent. These techniques allow for the examination of patients with renal impairment without the risk of a systemic nephrogenic fibrosis, while they also offer the possibility for repeated measurements. Diffusion weighted imaging (DWI) measures the Brownian motion of molecules as well as effects of blood microcirculation within a tissue and can be quantified by the calculation of ADC (apparent diffusion coefficient) maps [15]. Diffusivity of renal tissue is restricted when the interstitial space is narrowed, e.g., due to infiltration of inflammatory cells or fibrosis, which are associated with ADC reduction [14,16]. Mapping of the transverse relaxation time T2 allows for the quantification of water content within the renal tissue. Thus, a correlation of T2 relaxation times and tissue water content in the kidney has been reported in previous studies [17,18,19]. Therefore, an increase in the T2 relaxation times may be used as a marker for the extent of renal tissue edema.

In this study, we evaluated the benefit of two functional MRI techniques: Diffusion Weighted Imaging (DWI) and T2 mapping in the early phase after renal IRI for indication for progression to chronic kidney disease. MRI examinations were performed in B6 and CD1 mice that are known to have different susceptibilities to IRI. We hypothesized that CD1 mice, being more sensitive to IRI, display significant different data from functional MRI in the early phase after IRI.

## 2. Materials and Methods

### 2.1. Animals

All experiments were performed with male B6- and male CD1-mice (12–16 weeks, 20–30 g) purchased from Charles River (Sulzfeld, Germany). All animals received a standard diet with free access to tap water. All procedures were carried out according to guidelines from the German Society for Animal Science (Gesellschaft fuer Versuchstierkunde; GV-SOLAS) and were approved by the local animal protection committee (Niedersaechsisches Landesamt fuer Verbraucherschutz und Lebensmittelsicherheit (LAVES), approval number 11/0492). The German guidelines are in accordance with the National Institute of Health guidelines for animal welfare.

### 2.2. Renal Ischemia Reperfusion Injury

AKI was induced by transient unilateral clamping of the right renal pedicle for 35 min in B6- and CD1-mice as described previously [14]. Briefly, mice were anesthetized with isoflurane and received butorphanol (1 mg/kg s.c.) for analgesia prior to surgery. Median laparotomy was performed and a non-traumatic vascular clamp was applied unilateral to the renal pedicle for 35 min. After surgery, the mice were returned to their cages and monitored until they were fully awake.

### 2.3. Animal Sacrifice and Organ Harvesting

Animals were sacrificed in deep isoflurane anesthesia (4%) while total body perfusion with ice cold PBS via the left ventricle caused circulatory arrest. IRI kidneys as well as contralateral kidneys were fixed in 4% paraformaldehyde for immunohistochemistry, shock frozen in liquid nitrogen for western blotting or stored in RNA later (Thermo Fisher Scientific, Waltham, MA, USA) for qPCR analysis.

### 2.4. Functional MRI

MRI was performed on *n* = 10 B6- and *n* = 5 CD1-mice on d1 and d14 following IRI as described previously by Hueper et al. [14]. Briefly, a 7-Tesla animal scanner (Bruker, Pharmascan, Ettlingen, Germany) was used in combination with a one-channel circular polarized volume coil (Bruker, Ettlingen, Germany). Animals were anesthetized by isoflurane inhalation. Respiratory triggered, fat-saturated T2-weighted turbo spin echo (TSE) sequences were acquired in axial and coronal planes, covering both kidneys. The coronal plane was adjusted to the long axis of both kidneys. Scan parameters were: TR/TE = 1500/33 ms, averages = 2, matrix = 256 × 256, field of view = 35 × 35 mm^2^, slice thickness = 1 mm. For the measurement of the diffusivity, fat-saturated echo-planar diffusion-weighted sequences were acquired: TR/TE = 4000/22 ms, 7 b-values 0, 50, 100, 200, 300, 500, and 700 s/mm^2^, matrix 128 × 128, FOV 35 × 35 mm^2^, slice thickness 2 mm. For quantification of T2 relaxation times of the renal tissue, a multiecho turbo spin echo sequence was used with the following parameters: TR = 2000 ms/TE = 11, 22, 33 ms; matrix 256 × 256, field of view = 35 × 35 mm^2^, slice thickness 2 mm.

### 2.5. Image Analysis

Parameter maps of T2 relaxation time and ADC were calculated using a monoexponential fit. Mean T2-values and values of renal diffusion were determined separately for the ischemic and the non-ischemic kidney by one reader, who was blinded to the animal group identity. Regions of interest (ROI) were placed manually into the cortex and the outer stripe of the outer medulla (OSOM) of T2-maps and into the renal cortex and renal medulla on ADC maps since outer and inner medulla cannot be differentiated on ADC maps. Relative T2-and relative ADC-values were calculated as the percentage of T2-value and ADC-value of the ischemic and the control kidney (the contralateral, non-ischemic kidney on d1 served as control). The absolute values of T2 and ADC of the non-ischemic (control) kidney on day 1 are shown in the Supplemental Material (Table S1).

Total kidney volume was determined separately for each kidney by manual segmentation of axial T2-weighted images. For the analysis of T2- and ADC-values, regions-of-interest (ROI) were placed using Osirix software, version 11.0 (Pixmeo, Bernex, Switzerland).

### 2.6. Pro-Inflammatory Cytokine Expression

Total kidney tissue mRNA was isolated using RNeasy Mini Kit (Qiagen, Hilden, Germany). Subsequently, cDNA was synthetized with Prime Script Reverse Transcriptase reagent (Takara, Japan) from DNase-treated total RNA. qPCR was conducted on a LightCycler 96 (Roche, Penzberg, Germany) using SYBR Green primers. The following primers were used: Monocyte Chemoattractant Protein-1 (MCP-1, Qiagen, #QT00167832) and Interleukin-6 (IL-6, Qiagen, #QT00098875). Hypoxanthine phosphoribosyl transferase (HPRT, Qiagen, #QT00166768) was used as house keeper for normalization. All samples were measured in triplicates. Gene expression was calculated using the relative quantification method ΔΔCq. Five mice from each group were analyzed.

### 2.7. Renal Immunohistochemistry

The middle part of the kidney was immediately fixed in 4% paraformaldehyde (PFA) and embedded in paraffin. Immunohistochemistry was done on 2 µm paraffin sections using the following primary antibodies: fibronectin (Paesel and Lorei, Rheinberg, Germany) and type IV collagen (Southern Biotechnology, Birmingham, AL, USA). Antigen retrieval was achieved by incubating sections with trypsin for 15 min at 37 °C or by microwaving (15 min at 600 W). Primary antibodies were incubated for 60 min at room temperature in the dark. Alexa Fluor conjugated secondary antibodies were incubated for an additional 60 min in the dark in order to achieve fluorescent visualization of bound primary antibodies. Analysis was performed in a blinded manner using a Leica imaging microscope. The following scoring system was applied for fibronectin and collagen-4 deposition: 0 = no fibronectin/collagen-4 deposition; 1 = mild fibronectin/collagen-4 deposition, 2 = moderate fibronectin/collagen-4 deposition, 3 = severe fibronectin/collagen-4 deposition, 4 = very severe fibronectin/collagen-4 deposition.

### 2.8. ELISA to Measure Systemic Chemokine (C-X-C Motif) Ligand 13 Levels

Whole blood samples were drawn via retro-orbital venous plexus puncture in mild anesthesia with isoflurane and centrifuged at 4 °C for 5 min (10,000 rpm). Serum was stored at −80 °C. Serum chemokine (C-X-C motif) ligand 13 (CXCL13) levels were measured by ELISA (Quantikine Mouse CXCL13/BLC/BCA-1, Immunoassay catalog no. MCX130) using the Tecan spectra mini ELISA reader (Tecan, Crailsheim, Germany). CXCL13 levels were quantified by comparison with internal CXCL13 standards. CXCL13 ELISA was performed in *n* = 7 CD1 mice from each group and in *n* = 12–21 B6-mice on d1 from three independent experiments.

### 2.9. Statistical Analysis

Statistical analysis was performed using SPSS software version 25 (IBM, Armonk, NY, USA) and GraphPad Prism software version 5 (GraphPad, San Diego, CA, USA). Normal distribution of data was tested according to Kolmogorov–Smirnov tests and parametric (unpaired *t*-test, Pearson coefficient of correlation) or non-parametric tests (Wilcoxon rank sum test) were chosen accordingly. For the comparison of relative T2-values and relative renal diffusion of the two mouse strains, unpaired *t*-tests (for T2-values, diffusion values of the renal medulla and diffusion values of the renal cortex on d1) and Wilcoxon rank sum tests (diffusion values of the renal cortex on d14) were performed. Additionally, the correlation between relative T2-values and relative renal diffusion was evaluated with the Pearson coefficient of correlation. Multiple comparisons of normally distributed data were analyzed by one-way ANOVA and group means were compared using a Tukey’s post-hoc test. Non-parametric analyses for multiple comparison were performed using the Kruskal-Wallis test followed by a Dunn’s test.

*p*-values less than 0.05 were considered as an indication of a significant difference. Data are presented as mean ± standard error of the mean (SEM).

## 3. Results

### 3.1. Ischemia-Reperfusion Injury and Complications

No complications occurred during surgery and the follow-up period. Survival of the animals was 100% and there was no need for sacrifice for human endpoints.

### 3.2. Diffusivity of Renal Tissue Was More Restricted in CD1 than in B6 Mice

**Figure 1:**
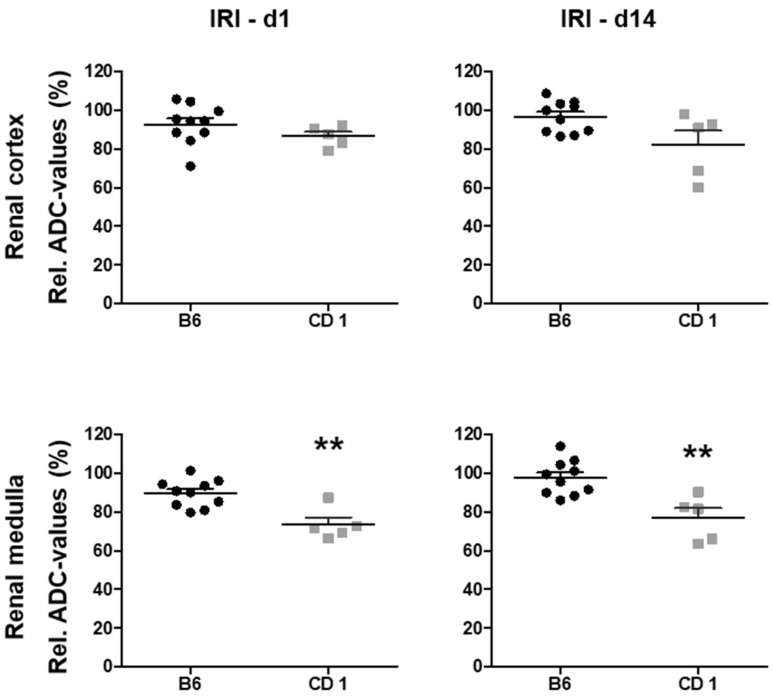
Diffusivity in the renal medulla was significantly more reduced in CD 1- mice compared to B6-mice on d1 and d14 after IRI. ADC-values were determined to quantify diffusivity in the renal cortex and renal medulla. Relative ADC-values were calculated as the percentage of ADC-values of the ischemic and the control kidney (the contralateral, non-ischemic kidney on d1 served as control). For the comparison of relative renal diffusion of the two mouse strains, un-paired *t*-tests (diffusion values of the renal cortex on d1, due gaussian distribution of data) and Wilcoxon rank sum tests (diffusion values of the renal cortex on d14, due to non-gaussian distribution of data) were performed. The presented dotplot diagrams show relative ADC-values of B6-mice (*n* = 10, black dots) and CD 1-mice (*n* = 5, grey squares) as well as mean and standard deviation. For both mouse strains, reduction of renal diffusivity can be detected after IRI. In the comparison of relative ADC-values of both mouse strains, relative ADC reduction is more pronounced in the renal medulla of CD 1-mice compared to B 6-mice on d1 and d14 after IRI. Diffusivity in the renal cortex was not significantly different between the two mouse strains. ADC = apparent diffusion coefficient, IRI = ischemia reperfusion injury, ** = *p* < 0.01.

Relative ADC values decreased on d1 after AKI in both mouse strains and remained at reduced levels two weeks after IRI (Figure 1). ADC reduction in the renal medulla was significantly more pronounced in CD1- compared to B6-mice on d1 (73 ± 3% vs. 90 ± 2%, *p* < 0.01) and on d14 (77 ± 5% vs. 98 ± 3%, *p* < 0.01). ADC reduction in the renal cortex showed the same trend, but was not significantly different on d1 (86 ± 2% vs. 93 ± 3%, ns) and d14 (82 ± 7% vs. 97 ± 3%, *p* = 0.01, Table 1).

### 3.3. Edema Formation Measured by T2 Mapping Was Pronounced in CD1

**Figure 2:**
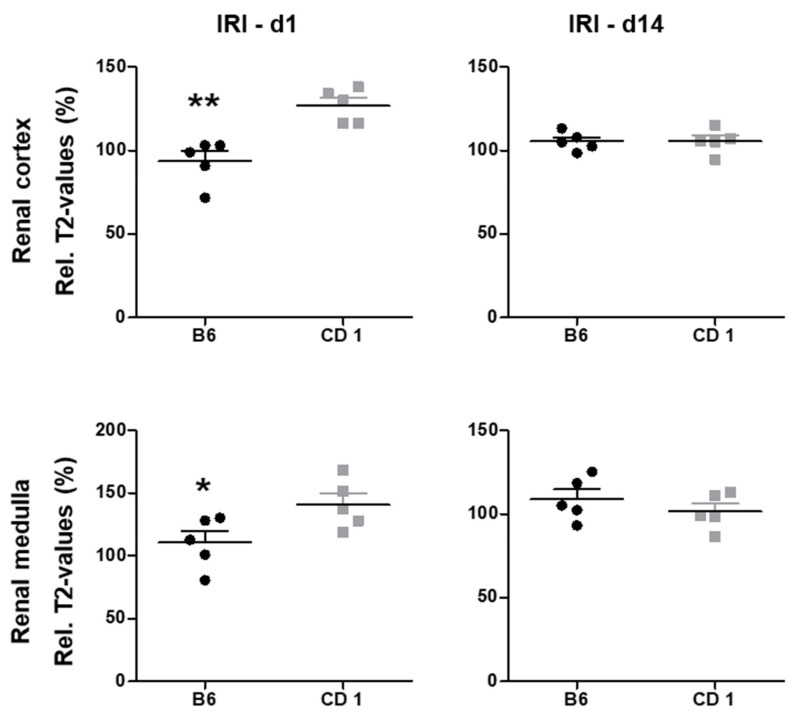
T2-values, representing tissue edema, were significantly more pronounced in CD1- mice on d1 after IRI. T2 maps were analysed to quantify tissue edema and relative T2-values were cal-culated as the percentage of T2-values of the ischemic and the control kidney (the con-tralateral, non-ischemic kidney on d1 served as control). For the comparison of rela-tive T2-values of the two mouse strains, unpaired *t*-tests (due to gaussian distribution of data) were performed. Relative T2-values of B6-mice (*n* = 5, black dots) and CD1-mice (*n* = 5, grey squares) are presented in dotplot diagrams with mean and standard devia-tion. T2-values were significantly higher in CD 1-mice on d1 in the renal cortex and OSOM compared to B6-mice, reflecting more pronounced tissue edema in these layers. No significant differences in tissue water content were found on d14 when using T2 mapping. IRI = ischemia reperfusion injury, * = *p* < 0.05, ** = *p* < 0.01.

**Figure 3:**
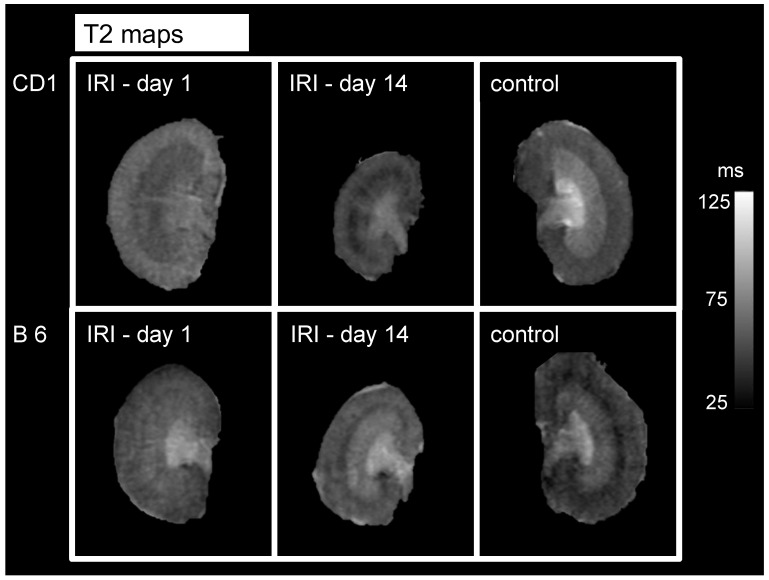
Examples of T2 maps of both mouse strains of the ischemic kidney on d1 and d14 after IRI as well as the control kidney (the contralateral, non-ischemic kidney on d1). T2-maps of the ischemic kidney of CD 1-mice (upper row) and B 6-mice (lower row) on d1 and d14 after unilateral clamping of the right renal pedicle for a duration of 35 minutes are depicted. The non-ischemic, contralateral kidney on d1 after surgery serves as an internal control. Window level and width as well as image size are similar for all parameter maps. Renal cortex and renal medulla of CD1-mice show more signal than B6 mice (reflecting more pronounced tissue edema). Note the reduction of kidney size on d14 in both mouse strains with accentuation in CD 1-mice. IRI = ischemia reperfusion injury, ms = milliseconds.

Tissue water content measured by T2 mapping was significantly higher in CD1-mice on d1 after IRI in the renal cortex (127 ± 5% vs. 94 ± 6%, *p* < 0.01) and the OSOM (141 ± 9% vs. 111 ± 9%, *p* < 0.05, Figure 2). 14 days after IRI, tissue water content was not significantly different between the two mouse strains. Relative T2-values are given in Table 2. Representative images are given in Figure 3.

### 3.4. Kidney Volume Loss Was More Pronounced in CD1, Compared to B6 Mice

IRI resulted in kidney volume loss after 2 weeks for both mouse strains. However, volume loss was greater in CD1- compared to B6-mice (50 ± 9% vs. 98 ± 6%, *p* < 0.001) indicating more pronounced irreversible kidney damage. No difference in organ volume was found at d1 after IRI (110 ± 8% vs. 120.5 ± 7%, *p* = 0.7, ns).

### 3.5. Functional MRI Parameters Correlate Significantly with Kidney Volume Loss

**Figure 4:**
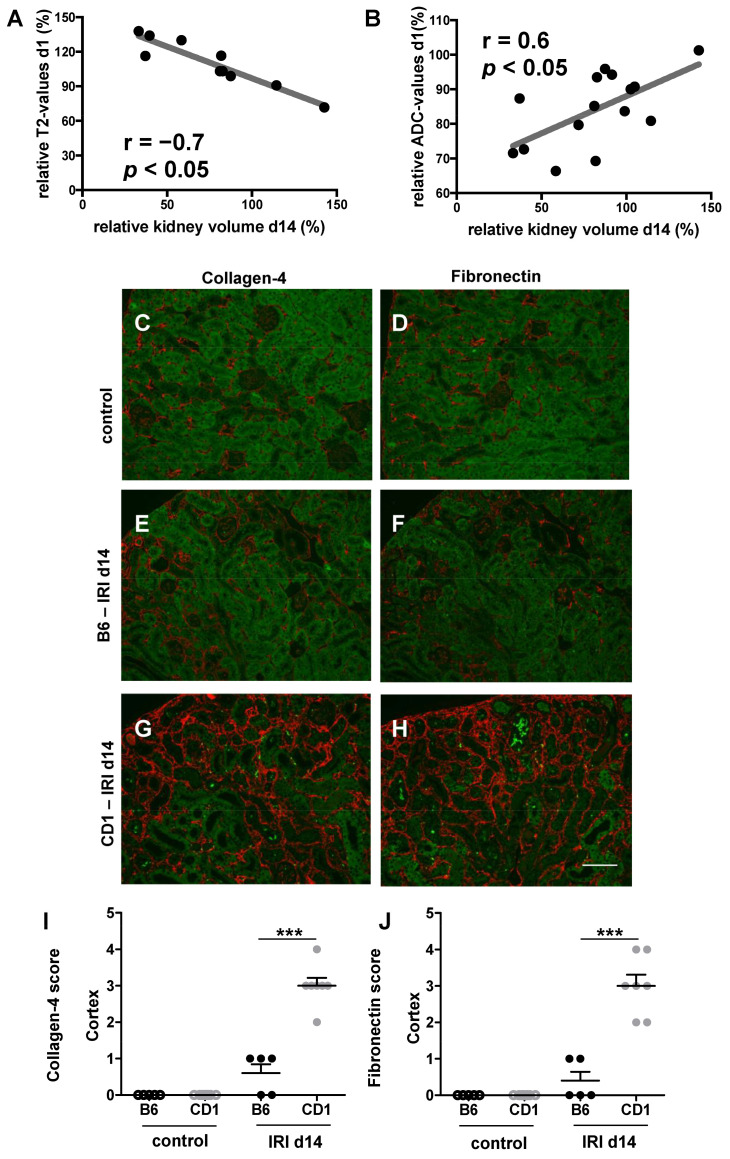
Kidney volume loss was found in both mouse strains 14 days after IRI. Pearson correlation of relative T2 values on d1 (**A**) and relative ADC values on d1 (**B**) are shown. Relative values of medullary ADC and of cortical T2 measured at d1 after surgery correlated significantly with kidney volume loss on d14. Collagen-4 expression (red staining. (**C**,**E**,**G**)) is pronounced in CD1 (**G**) compared to B6 (**E**) and control (**C**) two weeks after IRI indicating renal fibrosis. Moreover, fibronectin (red staining, (**D**,**F**,**H**)) as another marker of fibrosis was enhanced at d14 after IRI in CD1 (**H**) compared to B6 (**F**) and control (**D**). Semi-quantative histomorphological scoring of collagen-4 is shown in (**I**) and that of fibronectin in (**J**). The following scoring system has been used: 0 = no fibronectin/collagen-4 deposition; 1 = mild fibronectin/collagen-4 deposition, 2 = moderate fibronectin/collagen-4 deposition, 3 = severe fibronectin/collagen-4 deposition, 4 = very severe fibronectin/collagen-4 deposition. Five to seven mice each group were examined. One-way Anova followed by Tukey’s post-hoc test was used for multiple comparison of the normally distributed data. *** = *p* < 0.001. Scale bar = 100 µm.

Relative T2 values measured in the renal cortex on d1 correlated with relative kidney volume 2 weeks after IRI (r = −0.7, *p* < 0.05). Similarly, ADC values in the renal medulla measured on d1 correlated with relative kidney volume on d14 (r = 0.6, *p* < 0.05, Figure 4). Moreover, CD1 showed significantly increased renal fibronectin and collagen-4 expression at d 14 after IRI compared to B6 indicating progressive renal fibrosis in CD1 and only minor scarring in B6.

### 3.6. CD1 Exhibits Increased Inflammatory Response to IRI

**Figure 5:**
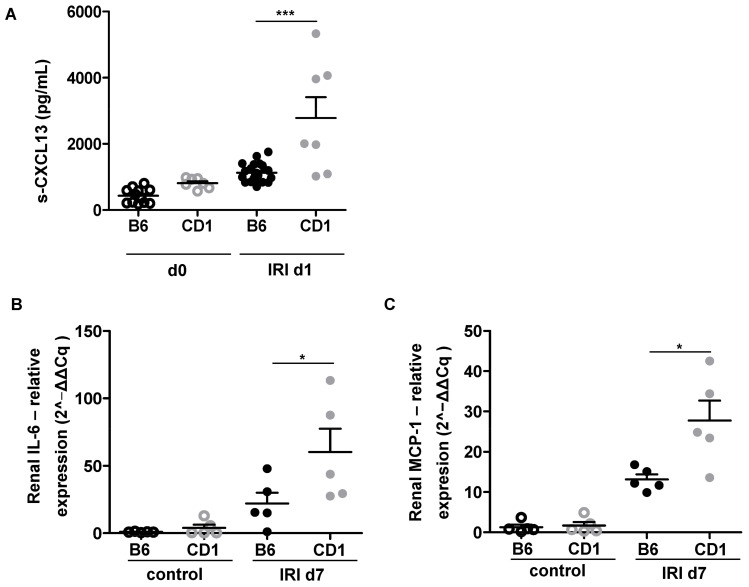
Renal inflammation was aggravated in CD1 after IRI. CD1 (grey dots) exhibits increased systemic upregulation of the B-cell chemoattractant CXCL13 on d1 after IRI compared to B6 (black dots) (**A**). Levels of the pro-inflammatory cytokines IL-6 (**B**) and MCP-1 (**C**) in the renal tissue were quantified by qPCR. Results were processed using the ΔΔCq method. CD-1 had significantly higher IL-6 (**B**) and MCP-1 levels (**C**) at d7 following renal IRI. CXCL13 ELISA was performed in *n* = 7 CD1 and *n* = 12–21 B6 mice from three independent experiments. qPCR was performed in *n* = 5 mice from each group. One-way Anova followed by Tukey’s post-hoc test was used for multiple comparison of the normally distributed data. * = *p* < 0.05 *** = *p* < 0.001.

Unilateral IRI resulted in systemic increase of CXCL13 as a chemoattractant for B-cells in CD1, but not B6 on d1 (Figure 5A) which was followed by enhanced local renal expression of the macrophage chemoattractant MCP-1 and the pro-inflammatory cytokine IL-6 in CD1 IR-kidneys on d7 (Figure 5B,C).

ADC-values were determined to quantify diffusivity in the renal cortex and renal medulla. Relative ADC-values were calculated as the percentage of ADC-values of the ischemic and the control kidney (the contralateral, non-ischemic kidney on day 1 served as control). For the comparison of relative renal diffusion of the two mouse strains, unpaired t-tests (diffusion values of the renal cortex on d1, due to Gaussian distribution of data) and Wilcoxon rank sum tests (diffusion values of the renal cortex on d14, due to non-Gaussian distribution of data) were performed. The presented dotplot diagrams show relative ADC-values of B6-mice (*n* = 10, black dots) and CD 1-mice (*n* = 5, grey squares) as well as mean and standard deviation. For both mouse strains, reduction of renal diffusivity can be detected after IRI. In the comparison of relative ADC-values of both mouse strains, relative ADC reduction is more pronounced in the renal medulla of CD 1-mice compared to B6-mice on d1 and d14 after IRI. Diffusivity in the renal cortex was not significantly different between the two mouse strains. ADC = apparent diffusion coefficient, IRI = ischemia reperfusion injury, ** = *p* < 0.01.

T2 maps were analyzed to quantify tissue edema and relative T2-values were calculated as the percentage of T2-values of the ischemic and the control kidney (the contralateral, non-ischemic kidney on d1 served as control). For the comparison of relative T2-values of the two mouse strains, unpaired *t*-tests (due to gaussian distribution of data) were performed. Relative T2-values of B6-mice (*n* = 5, black dots) and CD1-mice (*n* = 5, grey squares) are presented in dotplot diagrams with mean and standard deviation. T2-values were significantly higher in CD 1-mice on d1 in the renal cortex and OSOM compared to B6-mice, reflecting more pronounced tissue edema in these layers. No significant differences in tissue water content were found on d14 when using T2 mapping. IRI = ischemia reperfusion injury, * = *p* < 0.05, ** = *p* < 0.01.

T2-maps of the ischemic kidney of CD 1-mice (upper row) and B6-mice (lower row) on d1 and d14 after unilateral clamping of the right renal pedicle for a duration of 35 min are depicted. The non-ischemic, contralateral kidney on d1 after surgery serves as an internal control. Window level and width as well as image size are similar for all parameter maps. Renal cortex and renal medulla of CD1-mice show more signal than B6 mice (reflecting more pronounced tissue edema). Note the reduction of kidney size on d14 in both mouse strains with accentuation in CD1-mice. IRI = ischemia reperfusion injury, ms = milliseconds.

Pearson correlation of relative T2 values on d1 (A) and relative ADC values on d1 (B) are shown. Relative values of medullary ADC and of cortical T2 measured at d1 after surgery correlated significantly with kidney volume loss on d14. Collagen-4 expression (red staining. C, E, G,) is pronounced in CD1 (G) compared to B6 (E) and control (C) two weeks after IRI indicating renal fibrosis. Moreover, fibronectin (red staining, D, F, H) as another marker of fibrosis was enhanced at d14 after IRI in CD1 (H) compared to B6 (F) and control (D). 0 = no fibronectin/collagen-4 deposition; 1 = mild fibronectin/collagen-4 deposition, 2 = moderate fibronectin/collagen-4 deposition, 3 = severe fibronectin/collagen-4 deposition, 4 = very severe fibronectin/collagen-4 deposition. Five to seven mice each group were examined. One-way Anova followed by Tukey’s post-hoc test was used for multiple comparison of the normally distributed data. *** = *p* < 0.001.

## 4. Discussion

We could show that DWI and T2 mapping allow for the monitoring of pathophysiological changes after renal IRI. Tissue water content (such as edema) and infiltration of inflammatory cells (leading to a reduced diffusivity) are hallmarks of IRI and can be quantified by functional MRI. It has been demonstrated in a previous study that CD1 mice have an increased susceptibility to renal IRI compared to B6 mice [9]. In this study, we evaluated whether DWI and T2 mapping on functional MRI could detect morphological differences reflecting this different susceptibility. Indeed, we found that diffusion parameters were more restricted in CD1 mice at both time points. Tissue water content in the renal cortex and OSOM was more pronounced in CD1 mice in the early phase after AKI indicating increased edema formation. CD1-mice had greater kidney volume loss two weeks after IRI. Importantly, early ADC reduction and T2 relaxation time on d1 after IRI correlated with the subsequent kidney volume loss two weeks after IRI and could therefore serve as a predictive marker for the progression to CKD.

Diffusion weighted imaging with quantification of the ADC has been proven valuable for noninvasive characterization of renal pathology after AKI [14,20,21]. In a previous study, ADC correlated with kidney volume loss and was related to inflammatory cell infiltration and interstitial fibrosis [14]. In our study, ADC values were significantly more reduced in CD1-mice compared to B6-mice highlighting the value of these techniques in detecting the increased susceptibility to renal IRI of CD1. On a cellular basis, within 24 h after IRI, an inflammatory cascade is initiated [22] by continued production and release of chemokines and cytokines that further enhance local inflammatory response by attracting inflammatory cells. We found increased renal expression of the macrophage chemoattracant MCP-1, IL-6 and systemic upregulation of the B-cell chemoattractant CXCL13 after IRI in CD1. This is associated with ADC reduction in the renal medulla. This is in line with a previous study showing that a decrease of ADC after unilateral ureteral obstruction (UUO) was associated with infiltration of inflammatory cells and cell swelling, leading to narrowed interstitial space [15,16].

T2 mapping as a marker of renal tissue edema has been evaluated earlier and showed a linear relation to tissue water content [19], correlated with the severity of AKI and was related to inflammatory cell infiltration and kidney volume loss four weeks after IRI [14]. Elevation of T2 values might indicate tissue water content for both interstitial fluid (due to capillary leakage) and cell swelling [4]. Notably, DWI and T2 mapping have been applied in various renal injury models such as UUO [15,16] and IRI with different ischemia times [14]. Our work adds to the previous studies that ADC and T2 mapping at a very early time point after IRI, namely d1, can predict development of renal fibrosis already at d14. In a previous study, later time points for the correlation of ADC with kidney volume loss were used [14].

The early phase of AKI (1–3 days after kidney injury) is characterized by cellular injury, particularly in the renal tubular epithelial cells and vascular endothelial cells [23,24]. With this endothelial cell dysfunction, migration of inflammatory cells and vasopermeability is dysregulated resulting in cell infiltration and tissue edema and is most pronounced in the corticomedullary junction and outer medullary region [23]. CD1-mice showed a higher grade of tissue water content than B6-mice in the early phase following IRI (d1), as measured in the OSOM and the renal cortex. The later phase of AKI is known as the recovery phase (starting approximately 8–10 days after AKI) and is characterized by repair and differentiation of cells, re-establishment of epithelial polarity and the return to normal cellular and organ function. At that time point (d14), relative T2 values of the renal cortex and OSOM in both mouse strains returned almost completely to normal (Table 2, Figure 2 and Figure 3).

In a previous study on B6-mice, a unilateral IRI period of 35 min resulted in moderate AKI and kidneys were able to regenerate in B6-mice [25]. After a unilateral IRI period of 45 min (severe AKI), renal perfusion remained impaired until d28 after AKI, and a marked kidney volume loss was observed [25,26] indicating irreversible kidney damage. In our study, kidney volume loss at d14 was significantly more pronounced in CD1-mice with a relative kidney volume of 50% compared to B6-mice with a relative kidney volume of 98%. Histologically, marked fibronectin and collagen-4 disposition was evident in the ischemic kidneys of CD1 two weeks after IRI indicating renal fibrosis, which is the morphological correlate of kidney volume loss. Therefore, kidney volume loss after a 35 min-IRI in CD1-mice is comparable to kidney volume loss in B6-mice that was reported after 45 min-IRI with a relative volume of 60% [14]. Furthermore, ADC values and T2 values on d1 correlated significantly with kidney volume loss on d14 (Figure 4). Determination of these functional MRI parameters early after renal IRI might therefore be useful as predictive marker for progression to CKD. In a previous study, tubular epithelial cell proliferation early after renal IRI was present in B6 and absent in CD1. In addition, CD1-mice had increased rarefication of peritubular capillaries following IRI, which was associated with development of progressive renal fibrosis [9]. In this study, we showed that ADC and T2 values might be useful as predictive marker for progression to CKD.

Using diffusion weighted imaging and T2 mapping for longitudinal evaluation and quantification of changes that occur after renal IRI was feasible in our study. Functional MRI can be applied without the use of contrast agent and can be used to monitor changes over time. Furthermore, we were able to characterize diffusion restriction and water tissue content after IRI and find differences between the two mouse strains in vivo. Since no contrast media is required, this examination is also suitable for patients with kidney injury meaning that translation into clinical practice is possible. As noninvasive parameters, functional MRI can be used for longitudinal investigations such as monitoring of treatments and diseases. Since changes in functional MRI measurements can be detected early in the course of the disease when no kidney volume loss has occurred, it can help detect renal pathology early and may offer the possibility to modify treatment plans accordingly.

A limitation of our study is that we used the contralateral kidney of the same animal as a control. It has been reported earlier that the contralateral kidney increases in kidney volume 28 days after unilateral IRI by approximately 20% [25]. Therefore, the contralateral kidney on d1 after IRI served as control for both measurements. Still, the relative differences calculated in this study might underestimate the changes of the single kidney after IRI. Concerning DWI and T2 mapping, no major changes of the contralateral kidney have been reported over time [14]. Furthermore, we only used a monoexponential model to calculate ADC maps. The application of biexponential models might increase information from DWI concerning perfusion fraction and diffusion fraction [27].

## 5. Conclusions

In conclusion, DWI and T2 mapping allowed characterization of renal pathophysiology after IRI in mice and found differences between the two mouse strains. CD1-mice had higher susceptibility to IRI and subsequent progression to CKD. Changes in functional MR-parameters can be detected early after kidney injury and correlate with kidney volume loss. This predictive quality could also be useful in clinical settings. A translation of functional MRI into clinical practice could therefore be valuable and, due to its non-invasivity, imaginable.

## Figures and Tables

**Table 1:** Relative values of ADC maps at d1 and d14 after 35 min-IRI.

		d1		d14	
		**Mean ± SEM**	***p*-Value**	**Mean ± SEM**	***p*-Value**
cortex	CD 1	86 ± 2%	0.2, ns *	82 ± 7%	0.2, ns ^#^
	B6	93 ± 3%		97 ± 3%	
medulla	CD 1	73 ± 3%	<0.01 *	77 ± 5%	<0.01 *
	B6	90 ± 2%		98 ± 3%	

SEM = standard error of the mean, ns = not significant. * determined from unpaired *t*-tests, ^#^ determined from Wilcoxon rank sum tests.

**Table 2:** Relative values of T2 maps at d 1 and d14 after 35 min IRI.

		d1		d14	
		**Mean ± SEM**	***p*-Value**	**Mean ± SEM**	***p*-Value**
cortex	CD 1	127 ± 5 %	<0.01 *	106 ± 3%	1.0, ns *
	B6	94 ± 6 %		106 ± 3%	
OSOM	CD 1	141 ± 9%	<0.05 *	102 ± 5%	0.4, ns *
	B6	111 ± 9%		109 ± 6%	

OSOM = outer stripe of outer medulla, SEM = standard error of the mean, ns = not significant. * determined from unpaired *t*-test.

## Data Availability

The data presented in this study are available on request from the corresponding author.

## Supplementary Materials

The following are available online at https://www.mdpi.com/article/10.3390/biomedicines9081071/s1, Table S1: Absolute values of T2 and ADC of the non-ischemic (control) kidney on day 1.
