# Supplementary material for: Diffusion Weighted Imaging and T2 Mapping Detect Inflammatory Response in the Renal Tissue during Ischemia Induced Acute Kidney Injury in Different Mouse Strains and Predict Renal Outcome
Source: Biomedicines. 2021 Aug 23;9(8):1071. doi: 10.3390/biomedicines9081071 (PMC8393575; doi:10.3390/biomedicines9081071)
Supplement: Supplementary file 1 [file biomedicines-09-01071-s001.zip › biomedicines-1256529-supplementary.pdf]

Table S1. Absolute values of T2 and ADC of the non-ischemic (control) kidney on day 1.

Mouse strain	T2 values (ms)		ADC values (10^{-3} mm^2/s)	
	Cortex	OSOM	Cortex	Medulla
CD1	46.0±2.1	45.9±2.0	2.0±0.2	2.0±0.2
B6	47.3±3.0	44.7±5.1	1.6±0.1	1.6±0.2

Data is presented as mean±standard deviation. ADC=apparent diffusion coefficient.
